# Comparison of alternate scoring of variables on the performance of the frailty index

**DOI:** 10.1186/1471-2318-14-25

**Published:** 2014-02-24

**Authors:** Fernando G Peña, Olga Theou, Lindsay Wallace, Thomas D Brothers, Thomas M Gill, Evelyne A Gahbauer, Susan Kirkland, Arnold Mitnitski, Kenneth Rockwood

**Affiliations:** 1 Geriatric Medicine Research, Dalhousie University, Halifax, NS, Canada; 2 Department of Internal Medicine, Yale School of Medicine, New Haven, CT 06504, USA; 3 Department of Medicine, Dalhousie University, Halifax, NS, Canada; 4 Department of Community Health and Epidemiology, Dalhousie University, Halifax, NS, Canada; 5 Centre for Health Care of the Elderly, QEII Health Sciences Centre, Capital District Health Authority, Dalhousie University, Suite 1421, 5955 Veterans’ Memorial Lane, Halifax, NS B3H 2E1, Canada

**Keywords:** Aging, Frailty index, Deficit accumulation, Coding

## Abstract

**Background:**

The frailty index (FI) is used to measure the health status of ageing individuals. An FI is constructed as the proportion of deficits present in an individual out of the total number of age-related health variables considered. The purpose of this study was to systematically assess whether dichotomizing deficits included in an FI affects the information value of the whole index.

**Methods:**

Secondary analysis of three population-based longitudinal studies of community dwelling individuals: Nova Scotia Health Survey (NSHS, n = 3227 aged 18+), Survey of Health, Ageing and Retirement in Europe (SHARE, n = 37546 aged 50+), and Yale Precipitating Events Project (Yale-PEP, n = 754 aged 70+). For each dataset, we constructed two FIs from baseline data using the deficit accumulation approach. In each dataset, both FIs included the same variables (23 in NSHS, 70 in SHARE, 33 in Yale-PEP). One FI was constructed with only dichotomous values (marking presence or absence of a deficit); in the other FI, as many variables as possible were coded as ordinal (graded severity of a deficit). Participants in each study were followed for different durations (NSHS: 10 years, SHARE: 5 years, Yale PEP: 12 years).

**Results:**

Within each dataset, the difference in mean scores between the ordinal and dichotomous-only FIs ranged from 0 to 1.5 deficits. Their ability to predict mortality was identical; their absolute difference in area under the ROC curve ranged from 0.00 to 0.02, and their absolute difference between Cox Hazard Ratios ranged from 0.001 to 0.009.

**Conclusions:**

Analyses from three diverse datasets suggest that variables included in an FI can be coded either as dichotomous or ordinal, with negligible impact on the performance of the index in predicting mortality.

## Background

As individuals age, their vulnerability to adverse outcomes (including death) increases. Some individuals experience a state of increased vulnerability, known as frailty, which can be quantified using a frailty index [1]. The frailty index, introduced more than a decade ago [2], is a useful tool for assessing the health status of individuals, and for predicting an individual’s risk of adverse health outcomes [3,4]. Following a standard procedure, a frailty index can be constructed as the proportion of age-related health deficits an individual has accumulated [5]. Deficits can be any diseases, signs, symptoms, laboratory abnormalities, or functional or cognitive impairments, as long as about 30 measures are included which comprise a range of physiological systems. The more deficits one has, the higher their frailty index and the more vulnerable they are to adverse outcomes. Frailty indices demonstrate similar characteristics across diverse samples and settings, even when they employ different variables or different numbers of variables [1]. For example, frailty index values consistently increase with age, are strongly associated with mortality, and show higher values in women than in men.

Searle et al. [5] proposed criteria for selecting and coding health measures for inclusion as variables in a frailty index: deficits should be age-related, associated with adverse outcomes, contain little missing data (a 5% threshold was proposed) and not saturate with age (i.e. was not present in most people by some age, typically operationalized as being present in >80% of people by age 80). Searle and colleagues suggested that each variable included in a frailty index should be mapped to a 0 to 1 interval, assigned a value of 0 when a deficit is absent and 1 when it is fully expressed. It is not known, however, whether continuous or ordinal variables should be transformed into the dichotomous 0 and 1 values, or whether intermediate ordinal scores (e.g. self-rated health of “good” or “fair”), should be assigned intermediate values (e.g. 0.25 or 0.5). It is possible that by converting continuous variables (e.g. blood pressure) into dichotomous variables (e.g. “hypertension” present/absent), important information might be lost. The purpose of this study was to systematically assess whether dichotomizing deficits included in a frailty index affects the information value of the whole index. Within each of three large datasets with different settings and different durations of follow-up, we compared two different frailty indices using the same variables, but differing in whether the variables were dichotomized or categorized as ordinal variables. Specifically, we assessed for significant differences between: 1. descriptive characteristics of each frailty index, and; 2. its predictive validity using mortality as the primary outcome.

## Methods

This is a secondary analysis of three longitudinal studies: the Nova Scotia Health Survey (NSHS), the Survey of Health, Ageing and Retirement in Europe (SHARE), and the Yale Precipitating Events Project (Yale-PEP).

### Nova Scotia health survey

The Nova Scotia Health Survey began in 1995 and employed a representative probability sample designed by Statistics Canada. The sample included 3227 non-institutionalized Nova Scotians aged 18-99 (mean age = 48.1, SD = 19.8). There were approximately equal number of men and women in the sample (women n = 1618, 50.1%). Demographic, anthropometric, lifestyle, and risk factor data were collected at baseline and mortality data were obtained via linkage with the National Vital Statistics database 10 years following the baseline assessment. Full details of the data collection are presented elsewhere [6].

### The survey of health, ageing and retirement in Europe

The Survey of Health, Ageing and Retirement in Europe (SHARE) represents community-dwelling people aged 50 years and older across many European countries, and their spouses/partners [7]. Here, we included baseline data from the first two waves of SHARE (wave 1, 2004/2005: Austria, Belgium, Denmark, France, Germany, Greece, Israel, Italy, The Netherlands, Spain, Sweden, and Switzerland; wave 2, 2006/2007: Czech Republic, Poland, and Ireland) in which 37,546 individuals participated (mean age = 64.2, SD = 10.5 years; 56% female). We excluded spouses/partners under 50 years of age (n = 1,228). Mortality at 5 years was obtained from the second (2006/2007) and third waves (2008/2009) of SHARE for all countries except Israel and Ireland, where follow up data were not collected.

### The Yale precipitating events project

The Yale Precipitating Events Project (Yale-PEP) is a cohort study based in greater New Haven, Connecticut. Individuals were 70 years and older at the study’s inception (mean age = 78.4, SD = 5.3) [8,9]. The Yale-PEP survey contains longitudinal data of 754 community-dwelling, English-speaking, non-disabled persons who were not terminally ill. At baseline, most participants were women (n = 487, 64.6%), and the majority were white (n = 682, 90.5%), with a mean Mini-Mental State Examination (MMSE) [10] score of 26.8 (SD = 2.50). Death data were obtained from a follow up survey 155 months after baseline. Mortality is ascertained monthly and all deaths have been confirmed via death certificates [11].

### Frailty index

For each dataset, we constructed two frailty indices from baseline data following a standard procedure [5]. Within each dataset, one frailty index (FI_dichotomous_) was constructed from only dichotomous health variables (indicating the absence or presence of a deficit) and in the other frailty index (FI_ordinal_), as many variables as possible were ordinally coded (indicating the severity of a deficit, e.g. 0, 0.5, 1); both frailty indices in each dataset included the same variables and were constructed by the same member of our team. Across datasets, the frailty indices varied in the number of variables included, the proportion of variables that were only available as dichotomous measures, and in duration of follow-up. The FI_ordinal_ for NSHS included nineteen 2-level variables, two 3-level, one 4-level, and one 5-level variable (Total 23 variables, Table 1). The FI_ordinal_ for SHARE included sixty 2-level variables, one 3-level variable, two 4-level, and seven 5-level variables (Total 70 variables, Table 2). The FI_ordinal_ for Yale-PEP included twelve 2-level variables, fourteen 3-level variables, one 4-level, and six 5-level variables (Total 33 variables, Table 3). Scores on all frailty indices were calculated by dividing the sum of values on all included variables (deficits) out of the total number of non-missing variables. Participants missing >20% of data for variables in a frailty index were excluded for analysis of that frailty index [12].

**Table 1:** Coding of variables for the Nova Scotia Health Survey

**Variable name**	**Ordinal response/code**	**Dichotomous response/code**
Arthritis/Rheumatism	no = 0, yes = 1	no = 0, yes = 1
Back problems	no = 0, yes = 1	no = 0, yes = 1
Osteoporosis	no = 0, yes = 1	no = 0, yes = 1
Chronic bronchitis/Emphysema	no = 0, yes = 1	no = 0, yes = 1
Sinusitis	no = 0, yes = 1	no = 0, yes = 1
Cancer	no = 0, yes = 1	no = 0, yes = 1
Stomach/Intestinal ulcers	no = 0, yes = 1	no = 0, yes = 1
Urinary incontinence	no = 0, yes = 1	no = 0, yes = 1
Cataracts	no = 0, yes = 1	no = 0, yes = 1
Glaucoma	no = 0, yes = 1	no = 0, yes = 1
Hysterectomy/Oophorectomy	no = 0, yes = 1	no = 0, yes = 1
Mental illness	no = 0, yes = 1	no = 0, yes = 1
Other long-term condition	no = 0, yes = 1	no = 0, yes = 1
Need help with personal care	no = 0, yes = 1	no = 0, yes = 1
Need help with personal affairs	no = 0, yes = 1	no = 0, yes = 1
Confined to a bed or chair most of the day	no = 0, yes = 1	no = 0, yes = 1
Diabetes	no = 0, yes = 1	no = 0, yes = 1
PVD	no = 0, yes = 1	no = 0, yes = 1
Stroke	no = 0, yes = 1	no = 0, yes = 1
Able to go outside in good weather	yes without assistance = 0, yes with assistance = 0.5, no = 1	yes = 0, no = 1
Systolic blood pressure	90-140 = 0, <90 & 140-160 = 0.5, >160 = 1	90-140 = 0, <90 & > 140 = 1
LDL	<3.3 = 0, 3.3-4.1 = 0.333, 4.1-4.9 = 0.666, >4.9 = 1	<3.3 = 0, ≥3.3 = 1
Physical activity (3x weekly)	yes, for more than 6 months = 0, yes, for less than 6 months = 0.25, no, but intend to in the next 30 days = 0.5, no, but I intend to in the next 6 months = 0.75, no, and I do not intend to = 1	yes = 0, no = 1

**Table 2:** Coding of variables for the Survey of Health, Ageing and Retirement in Europe

**Variable name**	**Ordinal response/code**	**Dichotomous response/code**
Hospitalization in past year	no = 0, yes = 1	no = 0, yes = 1
Heart attack	no = 0, yes = 1	no = 0, yes = 1
Chronic lung disease	no = 0, yes = 1	no = 0, yes = 1
Osteoporosis	no = 0, yes = 1	no = 0, yes = 1
Parkinson disease	no = 0, yes = 1	no = 0, yes = 1
Stroke or CVD	no = 0, yes = 1	no = 0, yes = 1
Asthma	no = 0, yes = 1	no = 0, yes = 1
Cancer	no = 0, yes = 1	no = 0, yes = 1
Cataracts	no = 0, yes = 1	no = 0, yes = 1
High blood cholesterol	no = 0, yes = 1	no = 0, yes = 1
Long-term illness	no = 0, yes = 1	no = 0, yes = 1
High blood pressure	no = 0, yes = 1	no = 0, yes = 1
Diabetes or high blood sugar	no = 0, yes = 1	no = 0, yes = 1
Arthritis	no = 0, yes = 1	no = 0, yes = 1
Stomach or duodenal ulcer	no = 0, yes = 1	no = 0, yes = 1
Hip or femoral fracture	no = 0, yes = 1	no = 0, yes = 1
Heart trouble or angina	no = 0; yes = 1	no = 0; yes = 1
Falling down	no = 0; yes = 1	no = 0; yes = 1
Stomach or intestine problems	no = 0; yes = 1	no = 0; yes = 1
Difficulty biting on hard foods	no = 0, yes = 1	no = 0, yes = 1
Dizziness	no = 0, yes = 1	no = 0, yes = 1
Breathlessness	no = 0, yes = 1	no = 0, yes = 1
Sleeping problems	no = 0, yes = 1	no = 0, yes = 1
Incontinence	no = 0, yes = 1	no = 0, yes = 1
Swollen legs	no = 0, yes = 1	no = 0, yes = 1
Require dentures	no = 0, yes = 1	no = 0, yes = 1
Persistent cough	no = 0, yes = 1	no = 0, yes = 1
Pain in any joint	no = 0, yes = 1	no = 0, yes = 1
Climbing several flights of stairs	no = 0, yes = 1	no = 0, yes = 1
Pulling/pushing large objects	no = 0, yes = 1	no = 0, yes = 1
Dressing	no = 0, yes = 1	no = 0, yes = 1
Eating	no = 0, yes = 1	no = 0, yes = 1
Using a map to get around	no = 0, yes = 1	no = 0, yes = 1
Stooping/kneeling/crouching	no = 0, yes = 1	no = 0, yes = 1
Managing money	no = 0, yes = 1	no = 0, yes = 1
Lifting/carrying weights >5 kg	no = 0, yes = 1	no = 0, yes = 1
Getting in or out of bed	no = 0, yes = 1	no = 0, yes = 1
Walking across a room	no = 0, yes = 1	no = 0, yes = 1
Making telephone calls	no = 0, yes = 1	no = 0, yes = 1
Sitting for about two hours	no = 0, yes = 1	no = 0, yes = 1
Walking 100 meters	no = 0, yes = 1	no = 0, yes = 1
Getting up from a chair	no = 0, yes = 1	no = 0, yes = 1
Preparing a hot meal	no = 0, yes = 1	no = 0, yes = 1
Taking medications	no = 0, yes = 1	no = 0, yes = 1
Bathing or showering	no = 0, yes = 1	no = 0, yes = 1
Reaching or extending arms	no = 0, yes = 1	no = 0, yes = 1
Picking up a small coin from table	no = 0, yes = 1	no = 0, yes = 1
Using the toilet	no = 0, yes = 1	no = 0, yes = 1
Shopping for groceries	no = 0, yes = 1	no = 0, yes = 1
Doing work around house/garden	no = 0, yes = 1	no = 0, yes = 1
Appetite and eating	no = 0, yes = 1	no = 0, yes = 1
Suicidality	no = 0, yes = 1	no = 0, yes = 1
Lack of enjoyment	no = 0, yes = 1	no = 0, yes = 1
Trouble sleeping	no = 0, yes = 1	no = 0, yes = 1
Fatigue	no = 0, yes = 1	no = 0, yes = 1
Fear of falling down	no = 0, yes = 1	no = 0, yes = 1
Felt depressed	no = 0, yes = 1	no = 0, yes = 1
Pessimism	no = 0, yes = 1	no = 0, yes = 1
Interest	no = 0, yes = 1	no = 0, yes = 1
Concentration	no = 0, yes = 1	no = 0, yes = 1
Limitations with activities	not limited = 0, limited but not severely = 0.5, severely limited = 1	no limitations = 0, any limitations = 1
Frequency of vigorous activities	more than once a week = 0, once a week = 0.33, one to three times a week = 0.67, hardly ever, or never = 1	once a week or more = 0, less than once a week = 1
Frequency of moderate activities	more than once a week = 0, once a week = 0.33, one to three times a week = 0.67, hardly ever, or never = 1	once a week or more = 0, less than once a week = 1
Problems with eyesight	excellent = 0, very good = 0.25, good = 0.5, fair = 0.75, poor = 1	excellent/very good/good = 0 Fair/poor = 1
Hearing problems	excellent = 0, very good = 0.25, good = 0.5, fair = 0.75, poor = 1	excellent/very good/good = 0, fair/poor = 1
Self-rated health	excellent = 0, very good = 0.25, good = 0.5, fair = 0.75, poor = 1	excellent/very good/good = 0, fair/poor = 1
Orientation	4 correct responses = 0, 3 correct = 0.25, 2 correct = 0.5, 1 correct = 0.75, 0 correct = 1.0	3 or 4 correct = 0, fewer than 3 correct = 1
Mathematical performance	4 correct responses = 0, 3 correct = 0.25, 2 correct = 0.5, 1 correct = 0.75, 0 correct = 1.0	2 to 4 correct = 0, fewer than 2 correct =1
Delayed recall test	6 words or more recalled = 0, 5 words recalled = 0.25, 4 words = 0.5, 2 to 3 words = 0.25, 1 or 0 words = 1	3 words or more recalled = 0, fewer than 3 words recalled = 1
Verbal fluency score	25 or more animals named = 0, 21 to 24 named = 0.25, 17 to 20 named = 0.5, 13 to 16 named = 0.75, 12 or fewer named = 1	15 or more animals named = 0, fewer than 15 named = 1

**Table 3:** Coding of variables for the Yale Precipitating Events Project

**Variable name**	**Ordinal response/code**	**Dichotomous response/code**
Help eating	no = 0, yes = 1	no = 0, yes = 1
Help grooming	no = 0, yes = 1	no = 0, yes = 1
Help using toilet	no = 0, yes = 1	no = 0, yes = 1
Help up/down stairs	no = 0, yes = 1	no = 0, yes = 1
Help lifting 10 lbs.	no = 0, yes = 1	no = 0, yes = 1
Help shopping	no = 0, yes = 1	no = 0, yes = 1
Help with housework	no = 0, yes = 1	no = 0, yes = 1
Help with meal preparations	no = 0, yes = 1	no = 0, yes = 1
Help taking medication	no = 0, yes = 1	no = 0, yes = 1
Help with finances	no = 0, yes = 1	no = 0, yes = 1
Lost more than 10 pounds in the last year	no = 0, yes = 1	no = 0, yes = 1
High blood pressure	no = 0, yes = 1	no = 0, yes = 1
How health has changed in last year	better = 0, same = 0.5, worse = 1	better/same = 0, worse = 1
Heart attack	no = 0, suspected = 0.5, yes = 1	no = 0, yes/suspected = 1
CHF	no = 0, suspected = 0.5, yes = 1	no = 0, yes/suspected = 1
Stroke	no = 0, suspected = 0.5, yes = 1	no = 0, yes/suspected = 1
Cancer	no = 0, suspected = 0.5, yes = 1	no = 0, yes/suspected = 1
Diabetes	no = 0, suspected = 0.5, yes = 1	no = 0, yes/suspected = 1
Arthritis	no = 0, suspected = 0.5, yes = 1	no = 0, yes/suspected = 1
Chronic lung disease	no = 0, suspected = 0.5, yes = 1	no = 0, yes/suspected = 1
Feel everything is an effort	rarely = 0, sometimes = 0.5, most of the time = 1	rarely = 0, most/some of the time = 1
Feel depressed	rarely = 0, sometimes = 0.5, most of the time = 1	rarely = 0, most/some of the time = 1
Feel happy	most of the time = 0, sometimes = 0.5, rarely = 1	most/some of the time = 0, rarely = 1
Feel lonely	rarely = 0, sometimes = 0.5, most of the time = 1	rarely = 0, most/some of the time = 1
Have trouble getting going	rarely = 0, sometimes = 0.5, most of the time = 1	rarely = 0, most/some of the time = 1
BMI	18.5-24.99 = 0, <18.5 & 25-30 = 0.5, >30 = 1	18.5-24.99 = 0, <18.5 & > 25 = 1
Walk outside	often = 0, sometimes = 0.333, seldom = 0.666, never = 1	often = 0, sometimes/seldom/never = 1
Self-rating of health	excellent = 0, very good = 0.25, good = 0.5, fair = 0.75, poor = 1	good/very good/excellent = 0, fair/poor = 1
MMSE	>25 = 0, 21-24 = 0.25, 18-20 = 0.5, 10-17 = 0.75, <10 = 1	>24 = 0, <24 = 1
Usual pace	<9.3 = 0, 9.3-10.7 = 0.25, 10.8-12.6 = 0.5, 12.7-15.5 = 0.75, >15.5 = 1	≤16 = 0, >16 = 1
Rapid pace	<7.0 = 0, 7.0-8.4 = 0.25, 8.5-10.4 = 0.5, 10.5-12.9 = 0.75, >12.9 = 1	≤10 = 0, >10 = 1
Peak flow	Men	Men
	>526 = 0, 461-525 = 0.25, 408-460 = 0.5, 311-407 = 0.75, <310 = 1	>340 = 0, ≤340 = 1
	Women	Women
	>366 = 0, 324-366 = 0.25, 264-323 = 0.5, 210-263 = 0.75, <210 = 1	>310 = 0, ≤310 = 1
Shoulder strength	Men	Men
	>16.37 = 0, 14.57-16.36 = 0.25, 13.26-14.56 = 0.5, 10.83-13.25 = 0.75, <10.82 = 1	>12 = 0, ≤12 = 1
	Women	Women
	>13.00 = 1, 11.10-12.90 = 0.25, 9.40-11.00 = 0.5, 7.30-9.30 = 0.75, <7.29 = 1	>9 = 0, ≤9 = 1

### Statistical analysis

For each frailty index, we calculated mean scores (adjusted for sex) and tested the statistical significance of differences between the FI_ordinal_ and FI_dichotomous_ within each dataset using analyses of variance (ANOVA). To assess the impact of any differences between the FI_dichotomous_ and FI_ordinal_ for each dataset, we verified proportionality and performed multivariable Cox regression analyses for survival. The hazard ratios (HR) and 95% confidence intervals (CIs) for the two indices were adjusted for age and sex. Finally, we evaluated the difference in the ability of both FIs to predict mortality using Receiver Operating Characteristic (ROC) curves and compared the areas under the ROC curve. The statistical significance level was set to 0.05 and all calculations were performed using PASW18. Approval for the secondary analyses presented here came from the Research Ethics Committee of the Capital District Health Authority, Halifax, Nova Scotia, Canada.

## Results

**Figure 1:**
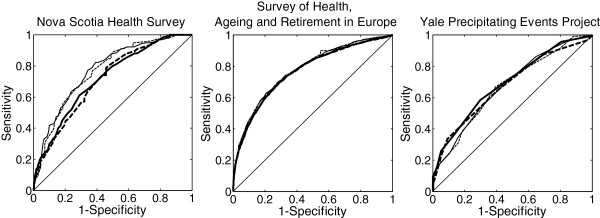
**The ability of the frailty index to predict mortality (ROC curves).** The ordinal (solid line) and dichotomous (segmented lines) frailty indices (FIs) are compared. Thick lines indicate males and thin lines females.

In SHARE and Yale-PEP, mean frailty index scores were significantly greater (p < 0.001) for the FI_ordinal_ compared with the FI_dichotomous_, whereas in NSHS the FI_dichotomous_ was greater (p < 0.001) (Table 4). The differences in mean scores between the two frailty indices from the same dataset were less than 0.02 in all datasets, which represents less than 1 deficit for NSHS and Yale-PEP and about 1.5 deficits for SHARE. The confidence intervals obtained from the Cox regression hazard ratios for FI_ordinal_ and FI_dichotomous_ overlapped (Table 5); absolute differences between the hazard ratios for the two FIs was 0.001 for SHARE, 0.008 for NSHS, and 0.009 for Yale-PEP. In each model, the age and sex covariates were also significant (p-value < 0.05). In Yale-PEP, 72.7% of the participants had died by 13 years follow-up, in NSHS 12.1% were deceased after 10 years, and in SHARE 11.7% had died by 5 years. The areas under the ROC curves for mortality prediction were the same for the FI_ordinal_ and FI_dichotomous_ in each dataset (Figure 1); their absolute differences ranged from 0 (SHARE, NSHS) to 0.02 (Yale-PEP) (Table 4). This pattern did not change when analyses were stratified by sex.

**Table 4:** Mean values and predictive ability of the frailty indices constructed with and without dichotomization of health deficits included in each frailty index

		Mean FI score* (SD)				**ROC area (CI)**		
**Survey**	**Male FI**_**ordinal**_	**Male FI**_**dichotomous**_	**Female FI**_**ordinal**_	**Female FI**_**dichotomous**_	**Male FI**_**ordinal**_	**Male FI**_**dichotomous**_	**Female FI**_**ordinal**_	**Female FI**_**dichotomous**_
NSHS	0.10 (0.08)	0.11 (0.08)	0.12 (0.10)	0.13 (0.11)	0.76 (0.73,0.80)	0.76 (0.73,0.79)	0.80 (0.77,0.84)	0.80 (0.77,0.83)
SHARE	0.16 (0.12)	0.13 (0.12)	0.19 (0.13)	0.17 (0.14)	0.77 (0.75,0.78)	0.77 (0.75,0.78)	0.78 (0.76,0.80)	0.78 (0.76,0.80)
Yale-PEP	0.23 (0.10)	0.21 (0.12)	0.26 (0.11)	0.25 (0.14)	0.70 (0.63,0.77)	0.69 (0.62,0.76)	0.68 (0.63,0.73)	0.66 (0.61,0.71)

*Significant differences between ordinal and dichotomous FIs for both males and females (p < 0.05).

SD = Standard Deviation, CI = Confidence Intervals, FI = frailty index.

SHARE = Survey of Health, Ageing and Retirement in Europe.

NSHS = Nova Scotia Health Survey.

Yale-PEP = Yale Precipitating Events Project.

**Table 5:** Cox proportional hazard ratios (HR) and 95% confidence intervals for time until death

**Survey**	**NSHS**		**SHARE**		**Yale-PEP**	
**FI-type**	**FI**_**ordinal**_	**FI**_**dichotomous**_	**FI**_**ordinal**_	**FI**_**dichotomous**_	**FI**_**ordinal**_	**FI**_**dichotomous**_
HR FI (CI)	1.04 (1.03,1.05)	1.03 (1.02,1.04)	1.04 (1.04,1.04)	1.04 (1.04,1.04)	1.04 (1.03,1.05)	1.03 (1.02,1.04)
HR age (CI)	1.11 (1.10,1.12)	1.11 (1.10,1.12)	1.08 (1.07,1.09)	1.08 (1.08,1.09)	1.08 (1.06,1.09)	1.08 (1.06,1.10)
HR sex (CI)	0.51 (0.41,0.62)	0.53 (0.42,0.65)	0.50 (0.45,0.55)	0.50 (0.45,0.55)	0.65 (0.54,0.79)	0.64 (0.53,0.77)
p-value FI	<10^-8^	<10^-8^	<10^-43^	<10^-43^	<10^-5^	<10^-5^
p-value age	<10^-8^	<10^-8^	<10^-43^	<10^-43^	<10^-5^	<10^-5^
p-value sex	<10^-8^	<10^-8^	<10^-43^	<10^-43^	<10^-5^	<10^-5^

CI = Confidence Intervals, FI = frailty index.

SHARE = Survey of Health, Ageing and Retirement in Europe.

NSHS = Nova Scotia Health Survey.

Yale-PEP = Yale Precipitating Events Project.

## Discussion

In this secondary analysis of data from the NSHS, SHARE, and Yale-PEP studies, we constructed two frailty indices for each dataset using the same variables but coding them differently; one included both dichotomous and ordinal variables (FI_ordinal_) whereas the other included only dichotomous variables (FI_dichotomous_). After comparing the ordinal and dichotomous frailty indices within each dataset, we found that their mean values and their ability to predict mortality were nearly identical. These findings, based on three diverse datasets, quantitatively confirm the flexible nature of the frailty index approach in relation to deficit variable coding. Our findings must be interpreted with caution. The samples from all three studies only included community-dwelling individuals. Our findings may not be generalizable to other populations such as institutionalized older adults and hospitalized patients. Even so, to maximize the generalizability of our results we included three diverse samples from different studies. The SHARE included Europeans age 50 and older with a follow up period of 5 years and Yale-PEP included Americans age 70 and older with a follow up period of 12 years. Note that the NSHS included Canadians age 18 and older with a follow up period of 10 years. In prior work we have calculated a frailty index for persons across the lifespan, starting at age 15 years [13]. It appears that the frailty index serves as a proxy measure of ageing [2,14,15]. This view (of the frailty index reflecting the deficit accumulation that drives mortality) has also been developed by other groups [16-18]. Other lines of evidence point to the importance of deficit accumulation prior to age 50 as a determinant of what happens in later life. For example, genes associated with greater longevity are typically associated with less deficit accumulation at younger ages [19] whereas states such as intellectual disability typically are associated with higher levels of deficit accumulation at younger ages (which nevertheless increase across the life course) [20]. Hence, there is a need to understand the impact that different scoring systems might have across the lifespan. Further, we chose to compare the predictive validity of the frailty indices using all-cause mortality at different lengths of follow-up. While the frailty index is not meant simply to predict mortality, all-cause mortality is useful here as it is dichotomous, easily verifiable and non-arbitrary.

In SHARE and Yale-PEP, the mean frailty index score was slightly higher for the FI_ordinal_ compared with the FI_dichotomous_, whereas in the NSHS the opposite was true. Even so, in all datasets the difference was minor, representing less than 1-1.5 items in the index. This is somewhat expected behavior for a frailty index. The criteria used to create a dichotomous variable from an ordinal one rely on the researcher (in this case, members of our research group), and it is expected that there may be differences between cutoff points among different researchers. However, this difference is expected to be minimal when at least 30 variables are included, and is expected not to be consistently higher or lower.

The mean frailty index scores differed across datasets, being highest in the oldest dataset (Yale-PEP) and lowest in the youngest (NSHS). Similarly, the mean score of the frailty indices and their predictive ability were different across datasets, but not between the two paired frailty indices within a dataset, which was the intended comparison. Across datasets, the risk associated with each increment in the frailty index crucially depends on the ambient or background level of risk, and so will differ. For this ambient risk, the outcomes of people with the lowest cores (e.g. frailty index = 0) can serve as an estimate [21].

## Conclusions

The frailty index provides a useful way to quantify the accumulation of relatively small health deficits across multiple physiological systems, and to identify and grade a state of overall vulnerability to outcomes. Based on our analysis of three diverse datasets we found that, if enough variables are included, dichotomizing variables or using them in ordinal form appears to have little impact on three important properties of the frailty index: the mean score, gender differences, and the ability to predict mortality.

## Abbreviations

FI: Frailty index; NSHS: Nova Scotia Health Survey; SHARE: Survey of Health, Ageing and Retirement of Europe; Yale-PEP: Yale Precipitating Events Project; ROC: Receiver operating characteristic; ANOVA: Analysis of variance; HR: Cox hazard ratio; CI: Confidence interval; SD: Standard Deviation; MMSE: Mini-Mental State Examination.

## Competing interests

KR and AM are applying for funding to commercialize a version of the Frailty Index based on a Comprehensive Geriatric Assessment. A company called Videx Canada has been incorporated for this. The version of the frailty index presented here is not the one that Videx aims to commercialize.

## Authors’ contributions

FP, OT, LW, TB conceived the article, conducted the analyses, and wrote the final draft. AM arranged for the SHARE data to be made available to our group and assisted with data analysis. KR contributed to the conception and initial design of the study, arranged funding, and revised previous drafts. TG and EG arranged for the Yale-PEP data to be made available to our group and revised previous drafts. SK arranged for the NSHS data to be made available to our group and revised previous drafts. All authors read and approved the final manuscript.

## Pre-publication history

The pre-publication history for this paper can be accessed here:

http://www.biomedcentral.com/1471-2318/14/25/prepub
